# Acknowledgment to Reviewers of *Membranes* in 2020

**DOI:** 10.3390/membranes11010068

**Published:** 2021-01-19

**Authors:** Membranes Editorial Office

**Affiliations:** MDPI AG, St. Alban-Anlage 66, 4052 Basel, Switzerland

Peer review is the driving force of journal development, and reviewers are gatekeepers who ensure that *Membranes* maintains its standards for the high quality of its published papers. Thanks to the cooperation of our reviewers, in 2020, the median time to first decision was 12 days and the median time to publication was 31 days. The editors would like to express their sincere gratitude to the following reviewers for their precious time and dedication, regardless of whether the papers were finally published:
Abatangelo, GiovanniLee, Kuo HaoAbbrent, SabinaLee, Pyung SooAbdala, AhmedLee, SungyunAbdel-Fattah, EssamLehocký, MariánAbdellatif, AbdelmohsanLei, WeiweiAbd-Elnaiem, AlaaLenik, JoannaAbejón, RicardoLi, ChengyongAbraham, NaderLi, HuiAbuin, GracielaLi, JianxinAchiou, BrahimLi, JunshengAdomavičiūtė, ErikaLi, Lu HuaAfsari, MortezaLi, ShibenAgarkov, DmitriiLi, WanbinAhmad, Mohd ZamidiLi, YuanchaoAirenne, Tomi T.Li, ZhipengAkhtar, SultanLi, ZidongAlaimo, AlessandroLiang, ShuangAlayande, Abayomi BabatundeLillepärg, JelenaAlbo, JonathanLin, HaiqingAlcoutlabi, MatazLin, HongjunAlexovič, MichalLin, ShuoAli, AamerLin, WeifengAli, GomaaLin, WeiyunAlique, DavidLin, XuboALjlil, Saad A.Linares, JoseAlkasrawi, MalekLing, ZhengAllegretti, ChiaraLipińska, AndreaAmissah, FelixLipnizki, FrankAmr, Abd El-Galil E.Lipscomb, GlennAn, ChunjiangLiu, BaijunAndersson, JakobLiu, BoAng, Micah Belle Marie YapLiu, DongxiaAnnesini, Maria CristinaLiu, GongpingAnokhina, Tatiana S.Liu, GuicaiAnsari, Mohamed AdamLiu, Hua-MinAntunes, Joana C.Liu, HuolongApollo, NicholasLiu, JunyiAppetecchi, Giovanni BattistaLiu, MinghuaArnusch, Christopher J.Liu, PengqingArtini, CristinaLiu, ShaominArunkumar, AbhiramLiu, Shyh-JiunArvanitis, AntoniosLiu, XianhuAshrafi, AmirmansoorLiu, YangAsif, Muhammad BilalLiu, YongAttia, Mohamed F.Lobo, AndersonAttri, PankajLong, RuiAwayda, MouhamedLópez, JulioAyyavoo, JayalakshmiLoulergue, PatrickAzhar, Muhammad RizwanLow, NicholasBacchin, PatriceLu, PengBae, Byung-ChanLu, WeiBagnato, GiuseppeLundin, Sean-Thomas B.Bagolini, AlviseLunguleasa, AurelBai, HongweiLuo, FabaoBaig, UmairLuo, TaoBakandritsos, AristidesMa, HongjuanBaldanzi, GianlucaMa, JiangyaBalme, SébastienMa, TianjiBan, YujieMa, XiaoliBanat, FawziMacedo, AntóniaBandini, SerenaMacedonio, FrancescaBao, YongmingMagalhães, LeandroBarelli, HélèneMagnacca, GiulianaBarloková, DankaMagnico, PierreBaschetti, Marco GiacintiMakisha, NikolayBaschir, LaurentiuMałolepszy, ArturBassin, Joao PauloMancini, MarcoBassyouni, MohamedMarchetti, LauraBayer, IlkerMaréchal, ManuelBazhenov, Stepan D.Mareev, SemyonBazzarelli, FabioMarquês, Joaquim Manuel TrigoBecker, MaikMartí-Calatayud, Manuel CésarBeckingham, BryanMartínez Rodrigo, JavierBekiari, VlasoulaMasoudi Soltani, SalmanBellanger, GilbertMatějka, MilanBelosludtsev, KonstantinMateus, MaríliaBelousov, Valery V.Matharu, Rupy KaurBento, FatimaMatko, VojkoBermeshev, MaximMatsumoto, MichiakiBernroider, GustavMatsuura, TakeshiBernuy-Lopez, CarlosMayyahi, Ahmed AlBi, LeiMažeikienė, AušraBiçer, YusufMazinani, SaeedBildyukevich, AlexandrMazor, OhadBin Darwish, NawafMazurkiewicz-Pawlicka, MartaBlandin, GaetanMazzei, RosalindaBobacka, JohanMazziotti Di Celso, GiuseppeBocharov, Eduard V.Melin, FredericBogacki, JanMelnikov, StanislavBohinc, KlemenMeng, FanbinBolotov, LeonidMeng, ShujuanBorisov, IlyaMeng, XiangchaoBostyn, StéphaneMihai, MaraBôta, AttilaMikhelson, KonstantinBounaceur, RodaMiller, Daniel N.Bramini, MattiaMinakshi, ManickamBraun, RalfMinamiki, TsukuruBreite, DanielMiranda, AdelaideBrown, Rhoderick E.Miranda, João MárioBruno, Nicholas C.Mitkowski, PiotrBruno, RosariaMizsey, PeterBryjak, MarekMoghaddam, SaeedBuchoux, SebastienMolina, SerenaBulgariu, LauraMolnar, OleksandrBusana, MattiaMondal, SayanButylskii, DmitriiMonjezi, Alireza AbbassiCabrales, LuisMonnot, MathiasCalabrò, FrancescoMonteiro, BernardoCalafel, ItxasoMontoro, CarmenCalvo, José IgnacioMorozan, AdinaCama, JehangirMorozova, SofiaCametti, CesareMoteki, TakahikoCannon, RichardMousa, HamoudaCao, BingMoutloali, RichardCao, BoMozo, Juan DanieCao, LiuyueMukherjee, MaitreyeeCarmela, ConidiMukhopadhyay, KausikCarraro, MauroMullaliu, AngeloCarreon, MoisesMüller, RüdigerCasado-Coterillo, ClaraMurmura, FedericaCassano, AlfredoMurmura, Maria AnnaCastro-Dominguez, BernardoMurphy, Eoin G.Cavallaro, GiuseppeMuschiol, JanCavayas, Yiorgos AlexandrosMuthumareeswaran, M.R.Cecconet, DanieleNabil, BouaziziCevik, EmreNagamine, KuniakiChae, Sung HoNagarkar, Amit A.Chakraborty, SudipNagul, Edward A.Chamberland, JulienNagy, EndreChang, HsuanNagy, MiklósCharfi, AmineNair, VaishakhChen, GeorgeNakahara, HiromichiChen, Yi-ChunNakielski, PawelChen, Yih-SharngNam, Sang YongChen, ZhitongNasir, RizwanCheong, HeesunNastase, FlorinChew, Nick Guan PinNatale, PaoloChew, Thiam LengNaumowicz, MonikaChiao, Yu-HsuanNechifor, GheorgheCho, KangwooNędzarek, ArkadiuszCho, YounghyunNemestóthy, NándorChoi, Jane RuNesterkina, MariiaChoi, Yong JunNeuman, ManuelaChu, XiangpingNevárez-Moorillón, Guadalupe VirginiaChuah, Chong YangNicoletta, Fiore PasqualeChung, Neal Tai-ShungNikolaeva, DariaChung, Sheng-HengNishihara, MasamichiChwojnowski, AndrzejNogueira, AntónioCiura, KrzesimirNomura, MikihiroClematis, DavideNtougias, SpyridonClodt, Juliana IsabelNuez, IgnacioCoduri, MauroNunna, Bharath BabuColli, Alejandro NicolásNyamutswa, Lavern T.Cortina, Jose LuisOchoa, Nelio ArielCosta, AnaOh, Hee JeungCosta, Carlos MiguelOh, Hyun-SukCowan, Matthew GreigOh, JonghyunCrespo, JoãoOhya, YoshikazuCretin, MarcOkamoto, YoshiyukiCrick, Colin R.Oliva, RosarioCruz-Alcalde, AlbertoOren, YoramCsanádi, JózsefOrfi, JamelCuccovia, Iolanda MideaOron, GideonCui, ZhaoliangOrooji, YasinCzernel, GrzegorzOrtiz Martínez, Víctor ManuelCzogalla, AleksanderOrtiz-Medina, JosueDarling, Seth B.Oumi, YasunoriDaumer, Marie LineOvalle-Encinia, OscarDavid, OanaPacheco Tanaka, David AlfredoDe Angelis, Maria GraziaPacheco, FedericoDe Filpo, GiovanniPadil, Vinod V. T.De Meijer, MariPadilla-Benavides, TeresitaDe Oliveira Lobo, AndersonPaganin, Valdecir AntonioDe Vos, Wiebe M.Paglieri, StephenDe Vries, Adrianus J.Pal, AnimeshDeimede, ValadoulaPalacio, LauraDema, RomanPalumbo, OrieleDeng, HuiningPanepinto, DeborahDeng, ShihaiPanglisch, StefanDerbal, KerroumPani, MarcellaDerbeli, MohamedPaolone, AnnalisaDeshpande, GauraviPapadakis, RaffaelloDi Bella, GaetanoPark, Jin YongDi Profio, GianlucaPark, Jin-SooDiddens, DiddoPark, TaehyunDiederichsen, KyleParnian, Mohammad JavadDing, BinParrino, FrancescoDing, YifuPasquini, LucaDinu, Maria ValentinaPatel, RajkumarDitl, PavelPatrauchan, Marianna A.Dmitrenko, Mariia E.Patsios, SotirisDo, Jing-ShanPawlowski, SylwinDobrzynski, PiotrPedersen-Bjergaard, StigDodero, AndreaPeer, PetraDołowy, KrzysztofPeng, HaoDong, DengpanPeng, XueyuanDong, LiangliangPeng, YongDong, WeibingPeng, YuanDong, XianpingPergher, SibeleDong, XuechengPernyeszi, TímeaDong, YingchaoPeters, ThijsDonzelli, ElisabettaPetriev, IliyaDos Reis, Rodrigo AzevedoPham, Chuyen VanDoutch, JamesPiotr, SzczepanskiDudek, GabrielaPirzada, TahiraDulova, NiinaPisk, JanaDumee, LudovicPismenskaya, NataliaDuskey, Jason ThomasPlisko, Tatiana V.Dutta, Ravi ChandraPoerio, TeresaEbrahimi, MehrdadPolańczyk, AndrzejEdomwonyi-Otu, Lawrence CPonomarev, Igor I.Eigenberger, GerhartPopovic, SvetlanaEinkauf, Jeffrey D.Post, JanEkielski, AdamPouliot, YvesEkwemalor, KingsleyPradanos, PedroEliseeva, TatianaPrasad, VikramEl-Naas, Muftah H.Prieto, ManuelElsaid, KhaledPrisic, SladjanaElSherbiny, Ibrahim M.A.Pruncu, Catalin I.Emeline, Perrier-GroultPulyalina, AlexandraEsche, ErikPuszkiel, JuliánEscorihuela, JorgeQanud, KhaledEsmaili, MansooreQasim, MuhammadEspíndola, Jonathan CawettiereQiang, YuhaoEsposito, ElisaQin, KairongEstay, HumbertoQin, YuEvangelopoulos, MichaelRaceanu, MirceaEwers, HelgeRadu, AleksandarFan, HongweiRafiq, SikanderFan, SenqingRahimpour, Mohammad RezaFarshchi Yazdi, Seyed Amir FouadRajca, MariolaFatyeyeva, KaterynaRamadoss, AnanthakumarFaustino, VeraRambabu, GutruFeng, HongboRao, SanjeevFeng, QichengRätzke, KlausFeng, ShilunReinosa, Julián JiménezFievet, PatrickReis, CristianoFijałkowski, KarolReis, Miria Hespanhol MirandaFilippov, AnatolyRestivo, JoãoFimbres Weihs, GustavoRider, Andrew N.Finke, Jan HenrikRincón, Marina E.Fiorenza, RobertoRobert, Philippe A.Firpo, GiuseppeRodrigues Reis, Cristiano E.Fischer, KristinaRodriguez De San Miguel, EduardoFlanagan, TedRonen, AvnerFologea, DanielRosenberger, SandraFontalvo, JavierRoubík, HynekFontananova, EnricaRoy, SagarFoti, ClaudiaRuan, XuehuaFraguas-Sánchez, Ana IsabelRuiz-García, AlejandroFrancesco, GalianoRuthstein, SharonFranco, Camilo AndresRybak, AleksandraFryczkowska, BeataSaakes, MichelFu, ZiaoSachdeva, Ir. SumitFunato, YosukeSadmani, A.H.M. AnwarFuoco, AlessioSaeki, DaisukeGaber, TimoSahoo, SanjubalaGalateanu, BiancaSamal, SangramGan, ShiyuSamhaber, Wolfgang M.Gao, ChunmeiSamsudin, Asep MuhamadGao, YanziSánchez Navarro, AmparoGarab, GyőzőSankarasubramanian, ShrihariGarcia, AndreinaSanthoshkumar, PutturGarcia-Bernabé, AbelSantoro, SergioGareev, Kamil Santos Juanes-Jorda, LucasGascooke, JasonSantos, Rafael M.Gazdzicki, PawelSan-Valero, PauGeffroy, Pierre-MarieSanyal, OishiGeisler, RamsiaSapalidis, AndreasGenduso, GiuseppeSavić, JelenaGeorgieva, RadostinaSawamura, Kentaro-ichiGeorgopanos, ProkopiosScarazzato, TatianaGhalei, BehnamScheepers, FabianGhica, Mihaela VioletaSchiraldi, ChiaraGhiorghita, Claudiu AugustinSchirhagl, RomanaGhoshal, ShraboniSchneck, EmanuelGiannotti, Marina InésSchuster, BernhardGierszewska, MagdalenaSchwantes, RebeccaGiuffre, OttaviaSeçkin, TurgayGizdavic-Nikolaidis, MarijaSeddighi, SadeghGlasmacher, BirgitSemino, RocioGlowinska, EwaSengupta, DebarunGoddard, AlanSeong, Jong GeunGoh, KunliSequeira, César A. C.Goh, Pei SeanSessa, LuciaGomez-Coma, LuciaSetničková, KaterinaGonzales, Ralph RollySevcsik, EvaGonzález, JoseSeverino, PatríciaGonzález-López, Tomás JoséSezgin, ErdincGościańska, JoannaSgreccia, EmanuelaGozálvez-Zafrilla, José MarcialShafieian, AbdellahGradov, Oleg M.Shaik, Mohammed RafiGrahovac, JovanaShao, JiahuiGreen, MatthewShao, LuGreen, MichaelSharma, Priyanka R.Grushevenko, EvgeniiaShaulsky, EvyatarGryta, MarekShellaiah, MuthaiahGrzegorzek, MartynaShen, JiangnanGu, QilinShevate, RahulGu, Zhi-GangShi, ZhengGuan, Kecheng Shimoga, GaneshGubler, LorenzShin, SehyunGueorguiev, Gueorgui K.Shtepliuk, IvanGuibal, EricSi, YangGuillén, ElenaSiekierka, AnnaGulari, ErdoganSikhwivhilu, KeneiloeGüler, EnverSillence, Dan J.Gulicovski, JelenaSilva, MónicaGumieniczek, AnnaSim, Lee NuangGurkan, BurcuSimari, CataldoGuskov, AlbertSinha Ray, SaikatGutierrez-Aguilar, ManuelSinha Ray, SumitGzara, LassaadSioutopoulos, DimitriosHabuda-Stanic, MirnaSivakumar, ManiHaddad, MaryamSkinner, JackHagar, MohamedSkorik, Yury A.Hamzah, Azrul AzlanSlouka, ZdenekHan, RunlinSnow, SamuelHan, XiuguoSoboloff, JonathanHanasoge, SrinivasSong, XiaoxiaoHandge, Ulrich A.Song, Young EunHarayama, TakeshiSousa, Sílvia A.Harja, MariaSoveral, GraçaHart, KyleStamatialis, DimitriosHartkamp, RemcoStaszak, MaciejHartman, MariuszStojmenović, MarijaHarun, Mohd HamzahStolarczyk, AgnieszkaHasan, Abdel Fattah R.Strandbakke, RagnarHashim, KhalidSu, BaoweiHashimoto, MasanoriSu, JincaiHassan, Mohammad K.Su, XiaoHassouna, Mohamed Salah El-DinSubach, Fedor V.He, WeiSubramanian, PalaniappanHe, ZemingSun, RamonHelmi, ArashSung, Gun YongHernández, AgustínSysel, PetrHernandez-Aldave, SandraSzaferski, WaldemarHeumann, RolfSze, Lai LiHidalgo, Asuncion MariaSzekely, GyorgyHirota, YuichiroSzeleszczuk, ŁukaszHlaibi, M.Szwast, MaciejHo, Jia ShinSzymański, KacperHofmann, AndreasTakamuku, ShogoHollmann, AxelTańczyk, MarekHomaeigohar, ShahinTang, KewenHong, Jin GiTang, Yuan-HuiHong, LiangTanioka, AkihikoHong, TaoTarditi, Ana MariaHorbett, Thomas ATéllez, CarlosHosui, AtsushiTeo, JeremyHou, JingweiTeodosiu, CarmenHou, ShangguoTeplyakov, VladimirHou, YiTeter, KenHsien, Tzu YangThakur, Vijay KumarHsu, Jyh-PingThekkiniath, JoseHu, AnmingThibault, JulesHu, ChengzhiTian, MiaoHu, JianTijing, LeonardHu, NingTiraferri, AlbertoHuang, GuoshengTiwale, NikhilHuang, ManhongTocci, ElenaHuertas, RosaToh, WilliamHyun, Dong ChoonTomašič, TihomirIfelebuegu, AugustineTong, XinIhsanullah, IhsanullahTorem, Maurício LeonardoIijima, KazutoshiTosheva, LubomiraIlavsky, JanToth, Andras JozsefIliev, BoyanTres, Marcus ViniciusImbrogno, AlessandraTringe, JosephInsepov, Zinetula (Zeke)Tripathy, Sunil KumarIorio, Ronald M.Trocino, StefanoIslam, Syed Z.Tsagarakis, KonstantinosIsmail, ManalTsehaye, Misgina TilahunItta, ArunTseng, Hui-hsinIvancev-Tumbas, IvanaTsuge, HideakiIvanets, AndreiTu, QingsongIwata, TomoyukiTufa, Ramato AshuJacomin, Anne-ClaireTundidor-Camba, AlainJanagam, Dileep R.Tung, Kuo-LunJansen, JohannesTuti, SimonettaJastrzab, RenataUchihashi, TakayukiJen, Tien ChienUrsino, ClaudiaJeon, Ju-WonUrtenov, MakhametJeong, EuikyoungVáclavíková, NatáliaJeong, KwanhoVaiano, VincenzoJeong, Sung-inValachová, KatarínaJho, Eun HeaVallabhajosyula, SaraschandraJi, GuozhaoVan Der Bruggen, BartJi, Yan-LiVan Goethem, CédricJia, DaVanangamudi, AnbharasiJia, HonggeVandezande, PieterJiang, ChenxiaoVardhan, HarshJiang, JunkeVasos, PaulJiang, Qiu-XingVersaci, MarioJiang, TaoVinothkannan, MohanrajJin, BoVisser, TymenJin, CongruiVlaicu, Ioana DorinaJiu, YamingVolkov, AlexeyJobin, Marie-LiseVolkov, VladimirJohnson, Colin P.Vranes, MilanJoo, JonghoonVrouwenvelder, JohannesJørgensen, Mads KoustrupWagh, PriyeshJoshi, RakeshWagh, Priyesh A.Jovanović, ZoranWaldron, KevinJue, Melinda L.Wang, Chih-MinJung, Eun SangWang, DavidKagramanov, GeorgiyWang, GuorongKahar, PrihardiWang, HongshengKalantari, KatayoonWang, JianKalantar-zadeh, KouroshWang, JieKaleekkal, Noel JacobWang, Jun (Jiangsu University of Science and Technology)Kallem, ParashuramWang, Jun (Nanjing University)Kallunki, TuulaWang, KeanKammakakam, IrshadWang, LeiKanematsu, HideyukiWang, LiguoKang, Byung-jaeWang, NaixinKang, Moon-sungWang, QiKang, Sang WookWang, TonghuaKang, Yang JunWang, XuefenKanno, TakahiroWang, YaomingKappert, EmielWang, ZhaoxuKarabagias, Ioannis K.Wang, ZhiKaravasili, ChristinaWang, ZhiweiKarimi, MohsenWang, ZhongpengKarkhanehchi, HamedWawrzkiewicz, MonikaKaruppasamy, K.Wei, Chun-haiKarvan, OguzWei, GangKashif, MuhammadWei, JunchaoKaškonienė, VilmaWei, YanyingKemperman, AntoineWei, ZhishunKertèsz, SzabolcsWeinman, Steven T.Khan, Mohammad EhtishamWeng, Guo-MingKhan, Mohammed NazeerWenten, I. GedeKharkar, PrathameshWieland, D. C. FlorianKikionis, StefanosWitt, KatarzynaKim, Ae RhanWłodarczyk, Paweł P.Kim, AlbertWong, Danny K. Y.Kim, HankiWu, BingKim, KeugtaeWu, Gwo-MeiKirichenko, KseniaWu, LiliKiss, EvaWu, Rome-MingKitamura, AkiraWu, YinghaoKitchen, PhilipWycisk, RyszardKlaysom, ChalidaWylie, Benjamin J.Koeppe, Roger E.Xiang, YuanKokosa, John M.Xiao, KangKolya, HaradhanXin, DongyueKong, XianXiong, XinKonieczkowska, JolantaXu, TaoKononova, Svetlana V.Xu, XinKoprowski, PiotrXu, ZhiweiKore, RajkumarXue, ShuangmeiKorolkov, Ilya V.Yamaki, TetsuyaKostoglou, MargaritisYan, HaiyangKoter, StanislawYan, KaiKothandaraman, JotheeswariYang, HuKoukounaras, AthanasiosYang, Ruey-JenKoutahzadeh, NeginYang, WeiKovacs, RenatoYin, Ben HangKovács, ZoltánYin, YanKowalczyk, TomaszYntema, DoekleKowalik-Klimczak, AnnaYoo, Dong JinKowalska, EwaYourey, WilliamKozmai, Anton EduardovichYu, DaweiKraslawski, AndrzejYu, HaifengKrieg, HenningYu, JaecheulKrumova, SashkaYu, Tzyy LeonKshatri, AravindYuan, ZhizhangKujawa, JoannaYuan, ZhongyunKujawski, WojciechYun, YanbinKulda, VlastimilYurekli, YilmazKulesha, OlgaZagklis, DimitrisKumar Thakur, VijayZarca, GabrielKurihara, MasaruZarybnicka, LucieKurlyandskaya, GalinaZarzycki, ArkadiuszKuroda, DaisukeZeng, LinKurtz, IraZhang, HongboKusuma, Victor A.Zhang, HuachengKuyukina, Maria S.Zhang, JunliangKvasha, AndriyZhang, LiqunKyriakopoulos, Grigorios L.Zhang, WenxiangLa Rosa, Angela D.Zhang, YajieLade, Harshad S.Zhang, YanlinLaguna-Bercero, Miguel ÁngelZhang, YizhouLai, Gwo-SungZhang, ZhongqiangLaín, SantiagoZhao, ShuaifeiLamas-Samanamud, GisellaZhao, YulongLameloise, Marie-LaureZheng, LibingLammertink, R.G.H.Zholobko, OksanaLanč, MarekZhou, JianLaoui, TaharZhou, TaoLasseuguette, ElsaZhou, XuLászló, ZsuzsannaZhou, ZhengzhongLatorrata, SaverioZhu, GuanghuiLau, Woei JyeZhu, JieLavric, VasileZhu, LingxiangLe, ZaiyuanZhu, MeihuaLe-Clech, PierreZhu, ShanLee, AlbertŽigon, JureLee, Eui-JongZolotukhin, Denis B.Lee, Hyun SuZoltan-Istvan, SzaboLee, Jae-SukZouboulis, AnastasiosLee, JaewooZuo, JianLee, Kew-Ho


